# Autoimmune-Associated Hemophagocytosis and Myelofibrosis in a Newly Diagnosed Lupus Patient: Case Report and Literature Review

**DOI:** 10.1155/2019/3879148

**Published:** 2019-01-09

**Authors:** Deonne Thaddeus V. Gauiran, Paula Victoria Catherine Y. Cheng, Christopher Ryan P. Pagaduan, Maria Clariza M. Santos

**Affiliations:** ^1^Section of Hematology, Department of Medicine, UP-Philippine General Hospital, Manila, Philippines; ^2^Department of Medicine, UP-Philippine General Hospital, Manila, Philippines; ^3^Section of Rheumatology, Department of Medicine, UP-Philippine General Hospital, Manila, Philippines

## Abstract

Bone marrow abnormalities in SLE are now becoming increasingly recognized, suggesting that the bone marrow may also be an important site of target organ damage. In this study, we present a rare case of concurrent autoimmune hemophagocytic syndrome and autoimmune myelofibrosis, potentially life-threatening conditions, in a newly diagnosed SLE patient. We report a case of a 30-year-old Filipino woman who presented with a one-year history of fever, constitutional symptoms, exertional dyspnea, joint pains, and alopecia and physical examination findings of fever, facial flushing, cervical lymphadenopathies, and knee joint effusions. Laboratory workup revealed pancytopenia with leukoerythroblastosis, elevated ESR, increased serum levels of transaminases, elevated CRP and LDH, hyperferritinemia, hypertriglyceridemia, proteinuria, hepatomegaly, and positive antinuclear antibody. Bone marrow aspiration and trephine biopsy revealed hemophagocytosis and moderate myelofibrosis. The patient was diagnosed with SLE with concomitant autoimmune-associated hemophagocytic syndrome and autoimmune myelofibrosis. Treatment with high-dose corticosteroids led to dramatic clinical improvement with normalization of laboratory data and complete resolution of bone marrow hemophagocytosis and myelofibrosis. Hemophagocytosis and myelofibrosis, although uncommon, are possible initial manifestations of SLE and should be included in the differential diagnosis of cytopenias in SLE. Thorough clinical assessment and microscopic bone marrow examination and timely initiation of corticosteroid therapy are essential in the diagnosis and management of these potentially life-threatening conditions. This case emphasizes that the bone marrow is an important site of target organ damage in SLE, and evaluation of cytopenias in SLE should take this into consideration.

## 1. Introduction

Hematologic abnormalities such as anemia, leukopenia, and thrombocytopenia are common in patients with systemic lupus erythematosus (SLE) [1]. The 2012 Systemic Lupus International Collaborating Clinics (SLICC) revised criteria include hemolytic anemia, leukopenia, and thrombocytopenia in its clinical criteria and positive direct antiglobulin test in its immunologic criteria [2]. Increased peripheral destruction of blood cells by circulating autoantibodies account for most hematologic manifestations of SLE [3]. Bone marrow abnormalities in SLE are now becoming increasingly recognized, suggesting that the bone marrow may also be an important site of target organ damage in SLE [4]. Here, we report a rare case of coexisting autoimmune hemophagocytic syndrome and autoimmune myelofibrosis, potentially life-threatening conditions, in a newly diagnosed SLE patient.

## 2. Case Report

A 30-year-old Filipino woman with no known comorbidities and good baseline functional capacity was admitted in our institution for a one-year history of intermittent high-grade fever (*T*_max_ 40°C), malaise, generalized weakness, anorexia, unintentional weight loss, alopecia, throat discomfort, exertional dyspnea, easy fatigability, and additive arthritis of bilateral knees, elbows, and small joints of both hands. Two months prior to admission, she consulted at a private clinic where a battery of tests showed negative Salmonella and dengue IgM and IgG, normal C-reactive protein, and normal rheumatoid factor. The patient does not smoke, drinks alcoholic beverages once a month, and denies illicit drug use. She previously worked as an adult entertainer in Japan during the early 2000s, during which she had more than 50 heterosexual partners. Her obstetric score is G2P2 (2002), with no previous fetomaternal complications. Family history is unremarkable.

On admission, she was noted to have tachycardia, fever (38.9°C), hyperemic conjunctivae, facial flushing, bilateral cervical lymphadenopathies, and arthritis of both knee joints. The initial working impression was fever of unknown origin with infection (tuberculosis and HIV), connective tissue disorder, and occult malignancy as major differential diagnoses.

Initial laboratory studies showed normocytic, normochromic anemia (hemoglobin 116 g/L), trace result on direct antiglobulin testing, and a 3.9 times elevated AST (145 U/L; reference range 15–37 U/L). Stool analysis was unremarkable, and fecal occult blood test was negative. Chest X-ray showed nonsignificant chest findings. Holoabdominal ultrasound revealed hepatomegaly with a liver span of 17.4 cm with smooth borders and a normal echo pattern but with a normal spleen. Multiple sets of sputum, stool, and urine acid-fast bacilli smears were all negative. Urinalysis showed minimal bacteriuria. ESR and CRP were both elevated. HIV antigen/antibody testing was negative. RPR, anti-HBs, anti-HCV, and HBsAg were all nonreactive. Three sets of blood cultures also turned out negative. Work-ups for infection and malignancy were unremarkable. She was initially started on piperacillin/tazobactam, omeprazole, tramadol, paracetamol, and naproxen. Naproxen was discontinued because of increase in facial flushing and periorbital edema.

**Figure 1:**
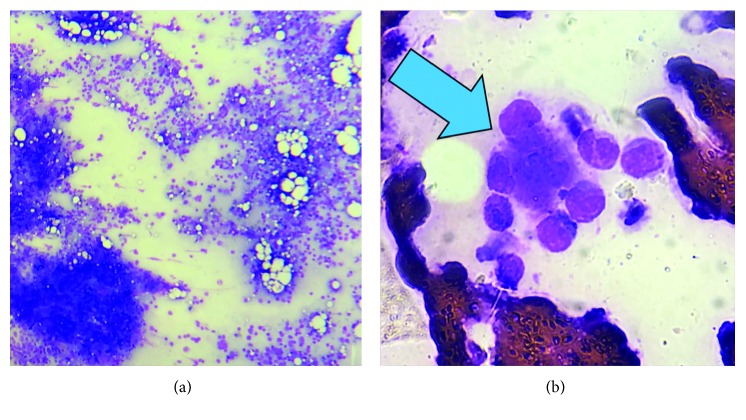
BMA smear. Normocellular marrow with trilineage hematopoiesis (a). Phagocytosis (blue arrow) by a histiocyte of erythroids and granulocytes (b).

**Figure 2:**
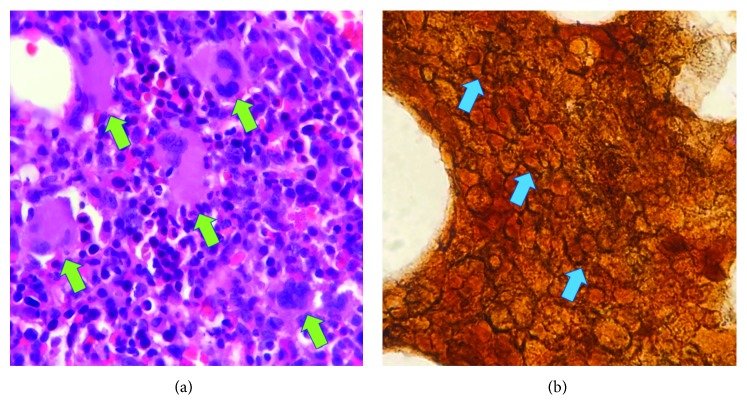
BM trephine biopsy. Normocellular marrow with megakaryocytic hyperplasia (small green arrows) in focal loose clusters (a). Reticulin staining showing moderate reticulin fibrosis (small blue arrows) (b).

On the 10^th^ hospital day, the patient was noted to have pancytopenia (hemoglobin 108 g/L, platelet count of 139 × 10^9^/L, WBC 3.18 × 10^9^/L) with leukoerythroblastosis. Laboratory testing during this time revealed an increase in ESR (from 47 to 92 mm/h), increase in AST (from 145 to 553 U/L), and an increase in ALT (from 32 to 189 U/L). LDH was noted to be markedly elevated (9680 U/L; reference range 100–190 U/L). Triglycerides (3.26 mmol/L) and serum ferritin (>4500 pmol/L) were also markedly elevated. 24-hour urine collection showed total protein of 900 mg with a total volume of 1500 mL. Repeat direct and indirect antiglobulin tests were negative. Anti–cyclic citrullinated peptide was negative. Antinuclear antibody was positive but anti-double-stranded DNA was negative, and C3 was normal. Bone marrow aspiration (BMA), and biopsy was done. The BMA smear revealed a normocellular marrow with trilineage hematopoiesis and the presence of hemophagocytosis (see Figure 1). The bone marrow trephine biopsy revealed a normocellular marrow with megakaryocytic hyperplasia with moderate myelofibrosis (WHO MF Grade 2) (see Figure 2). The bone marrow acid-fast bacilli smear was negative.

Having satisfied the 2012 SLICC revised diagnostic criteria, she was then diagnosed as a case of systemic lupus erythematosus with concomitant autoimmune-associated hemophagocytic syndrome (HScore of 229 with 97.8% probability of HLH) and autoimmune myelofibrosis. She was then started on intravenous hydrocortisone (prednisone 1 mkd equivalent), hydroxychloroquine, and calcium plus vitamin D supplements. During treatment, all clinical findings (e.g., malaise, generalized weakness, throat discomfort, fever, facial flushing, lymphadenopathies, and arthritis) and laboratory data (e.g., blood counts, LDH, and liver enzymes) gradually improved. No significant treatment-related side effects were noted.

**Figure 3:**
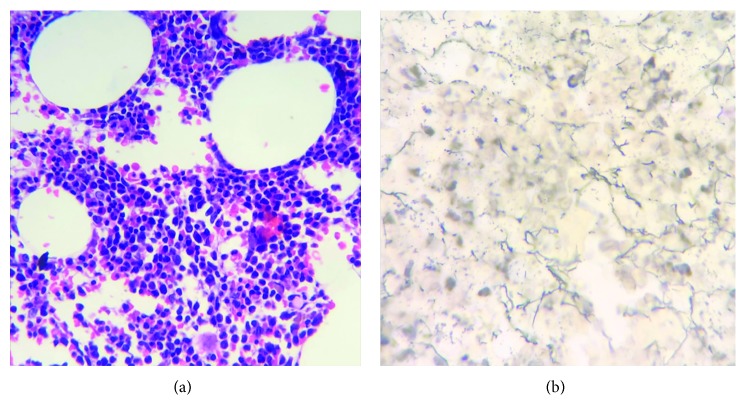
BM trephine biopsy. Normocellular marrow with trilineage hematopoiesis (a). Reticulin staining showing no significant fibrosis (b).

She was discharged on the 19^th^ hospital day on oral prednisone (1 mkd), hydroxychloroquine, and calcium plus vitamin D. She was closely followed at the outpatient clinics with note of continuous improvement in symptoms and laboratory findings. Prednisone was slowly tapered down. Hydroxychloroquine and calcium plus vitamin D were continued. Good adherence to treatment plan was noted. No significant therapy-related side effects were reported by the patient. A repeat bone marrow biopsy was done four months after discharge, which revealed normocellular marrow with trilineage hematopoiesis with complete resolution of myelofibrosis and hemophagocytosis (Figure 3).

## 3. Discussion

Hemophagocytic lymphohistiocytosis (HLH), also known as hemophagocytic syndrome (HS), is a multisystemic inflammatory syndrome brought about by secondary immune dysregulation [5–7]. NK cells and cytotoxic T cells in HLH fail to control activated macrophages resulting in excessive macrophage activity [6, 8]. Activated macrophages engulf mature and hematopoietic stem cells leading to cytopenias [9]. The subsequent overwhelming release of inflammatory cytokines also produces further suppression of hematopoiesis and causes the symptoms associated with the condition. It is usually characterized by fever (≥7 days, ≥38.5°C), splenomegaly, neurologic symptoms, lymphadenopathy, jaundice, edema, cytopenias, hypertriglyceridemia, hyperferritinemia, hypofibrinogenemia, coagulopathy, elevated serum transaminases, hypoalbuminemia, hyponatremia, and hemophagocytosis on liver, lymph node, or bone marrow histopathologic studies [5–7].

HLH can occur as a genetic or an acquired disorder [6, 7, 10, 11]. Genetic HLH, which is usually seen in the pediatric population and has an autosomal recessive or X-linked pattern of inheritance, can be further subdivided into the primary/familial form associated with FHL1, PFR1, UNC13D, STX11, and STXBP2 mutations and immune deficiency syndromes such as Chédiak–Higashi syndrome, type 2 Griscelli Syndrome, and X-linked lymphoproliferative syndrome [7, 12, 13]. Acquired HLH, in contrast to the genetic form, can occur at any age [7, 13]. It results from intense immunologic activation that may be brought about by severe infections, malignancies, autoimmune disorders, lymphoma and other malignancies, use of anticonvulsant medications, or immune suppression in the setting of stem cell transplantation [7, 10, 14].

The HLH Study Group of the Histiocyte Society released a diagnostic guideline for HLH in 2004, also known as the HLH-2004 diagnostic criteria [13]. According to the said criteria, at least five of the following criteria must be fulfilled for the diagnosis of HLH: fever; splenomegaly; cytopenias affecting two or three lineages (hemoglobin <90 g/L, platelet counts <100 × 10^9^/L, neutrophils <1.0 × 10^9^/L); hypertriglyceridemia (>3.0 mmol/L) and/or hypofibrinogenemia (<1.5 g/L); hemophagocytosis in bone marrow, spleen, or lymph nodes; hyperferritinemia (>1123 pmol/L); low or absent NK cell activity; and elevated level of sIL-2R (sCD25 > 2400 U/ml) [13]. Unfortunately, these diagnostic criteria were developed for the familial form of HLH and have never been validated in adults nor in acquired HLH [13, 15]. Moreover, some of the tests included in the criteria are not readily available, limiting its usefulness especially because the life-threatening nature of HLH requires timely diagnosis and initiation of treatment [7, 15]. To answer these limitations, Fardet et al. developed and validated a scoring system for HLH especially for the acquired form, the HScore [15]. It includes nine parameters with different scoring weights (underlying immunosuppression, maximal temperature, hepatomegaly, splenomegaly, hemoglobin, leukocyte count, platelet count, serum ferritin level, serum triglyceride level, fibrinogen level, AST, and hemophagocytosis on bone marrow aspirate), the sum of which produces a composite score with a corresponding probability of HLH [15]. The HScore is easily accessed and calculated using the following website: http://saintantoine.aphp.fr/score/. Using the HScore on our patient, we obtained a score of 229 with a 97.8% probability of HLH.

Autoimmune-associated hemophagocytosis syndrome (AAHS), also known as macrophage activation syndrome (MAS), is a subset of acquired HLH. It has been reported in cases of systemic juvenile idiopathic arthritis, adult-onset Still's disease (AOSD), systemic lupus erythematosus (SLE), rheumatoid arthritis (RA), Sjogren's syndrome, dermatomyositis, mixed connective tissue disease, systemic sclerosis, and Kawasaki disease [7]. AAHS in SLE is rare and reported only in 0.9% to 4.6% of patients diagnosed with SLE [16–18]. It commonly affects females with a median age of 43 years [16]. The mortality rate of AAHS ranges from 12.9% to 38.5%, emphasizing the need for early recognition and treatment. [19, 20]. AAHS may demonstrate all the clinical and laboratory features of HLH [7]. Reviews have shown, however, that coagulopathy and cardiac dysfunction are more common in AAHS and neurologic symptoms are more often progressive [21]. It must be underlined that not all patients with AAHS fulfill diagnostic criteria for HLH (HLH-2004) on presentation, thus, making the HScore a feasible option for this subset of patients suspected to have HLH [7, 21]. Another aid in clinching the diagnosis of AAHS, presented by Grom and Mellins, includes the following: persistently active underlying rheumatologic disease, fall in ESR, fall in platelet count, persistently high CRP, and increasing levels of serum D-dimer and ferritin [22]. Assessment of serum levels of sIL-2R and sCD163 may also help in the diagnosis of AAHS as higher levels of these are more suggestive of AAHS rather than classic/familial HLH [7, 22]. They may also be particularly helpful for patients with normal bone marrow biopsies [7, 22]. Nonetheless, a high index of suspicion is very important in the diagnosis of AAHS. The absence of active infection or malignancy, the presence of SLE in activity, and a very high HScore led us to diagnose the patient with AAHS.

Currently, there are no randomized controlled trials available to guide HLH treatments in adults [23]. Likewise, the treatment of AAHS from SLE is also not well established [24, 25]. In contrast to other causes of HLH in which the current treatment recommendation is 8-week induction with 150 mg/m^2^ etoposide and dexamethasone, patients with AAHS should be treated initially with parenteral steroids [23, 26]. In the absence of an adequate response, additional disease-specific immunosuppressive treatment is recommended [23]. Options include intravenous immunoglobulins (especially if there is an associated viral infection), cyclophosphamide, cyclosporine, tacrolimus, infliximab, and rituximab [23]. Gastroprotection, antibacterial, and antifungal prophylaxis are likewise recommended [23]. Positive treatment response is signified by clinical improvements in splenomegaly, lymphadenopathy, and fever and by laboratory improvements in serum levels of ferritin, liver function tests, fibrinogen, and sIL-2R [23]. Fortunately, institution of corticosteroids in our patient led to dramatic clinical and laboratory improvement, obviating the need for further immunosuppressive therapy.

Autoimmune myelofibrosis (AIMF), introduced by Paquette et al. in 1994, is a distinct clinicopathologic condition that occasionally occurs with autoimmune disorders such as SLE and RA [27]. Nonmalignant marrow fibrosis which occurs in the presence of a defined systemic autoimmune disease such as SLE or RA is termed secondary AIMF [28]. Primary AIMF, on the contrary, is a clinical condition with similar presentation to secondary AIMF but occurs in patients without a defined autoimmune disease [29]. It is imperative to differentiate AIMF from primary myelofibrosis (PMF), a clonal myeloproliferative neoplasm, because their clinical course, prognosis, and treatment vary markedly [30]. Bone marrow findings in AIMF include the following: variable bone marrow cellularity with reticulin and/or collagen fibrosis; absence of megakaryocytic, myeloid, or erythroid dysplasia; presence of lymphoid aggregates; and lack of osteosclerosis [28, 30, 31]. In contrast, PMF is characterized by bone marrow findings of moderate-to-severe reticulin fibrosis and the presence of megakaryocytic proliferation and atypia [32]. Cytopenias, leukoerythroblastosis, and presence of teardrop poikilocytosis may be seen in the peripheral blood of both AIMF and PMF [28, 33]. However, eosinophilia and basophilia are not features of AIMF [28, 29, 34]. The presence of autoantibodies completes the clinicopathologic pattern of AIMF; however, they may also be detected in some patients with PMF [28–30, 33, 34]. Clonal markers such as mutations in JAK2, CALR, and MPL are seen in PMF but are absent in AIMF [28, 30, 32]. Splenomegaly in AIMF is not usual and when present may only be minimal, in contrast with PMF in which palpable splenomegaly is an important feature [28, 29, 32].

The pathogenesis of AIMF is still not well-elucidated [31, 35]. The proinflammatory state seen in AIMF is postulated to stimulate nonclonal fibroblasts to produce excessive amounts of extracellular matrix resulting in the development of fibrosis [30]. Reactive cells in the marrow are thought to release growth factors known to induce collagen synthesis such as platelet-derived growth factor (PDGF), transforming growth factor beta (TGF-*β*), and epidermal growth factor [35, 36]. In addition, vasculitis-related bone marrow injury may stimulate fibroblast proliferation and activation [37]. Finally, binding of immune complexes to megakaryocyte Fc receptors may lead to excess release of PDGF, leading to fibrosis [35, 37].

A review of cases of SLE-related AIMF done by Del Porto et al. in 2018 revealed only 46 published cases globally [35]. Unlike PMF, which occurs most commonly in the sixth to seventh decades of life, the median age of patients with SLE-related AIMF is 36 years old (range of 12–70 years) [35, 38]. There is 1 : 6.5 male-to-female ratio in contrast to PMF, which affects men and women nearly equally [35, 38]. AIMF was noted to occur mainly in patients with active disease [35]. AIMF shows a more favourable course than PMF with reported mortality of only 20% (9/45 reported cases) [35]. In another cross-sectional study involving 41 patients with SLE presenting with cytopenias done by Wanitpongpun et al. in 2012, it was noted the myelofibrosis is an uncommon finding among patients with SLE even with low peripheral blood counts [39].

It is important to note that AIMF should be considered in cases of myelofibrosis in the presence of the following atypical features: young age, female sex, absence of splenomegaly, or absence of the JAK2 V617F mutation [4]. The presence of grade 2 reticulin fibrosis with no significant marrow dysplasia in a young, female patient with no splenomegaly and with active SLE led us to diagnose our patient with AIMF. Unfortunately, JAK2 mutation testing was not done due to financial constraints.

In contrast with PMF, AIMF is steroid-responsive [4, 35]. Review of the literature showed that 80% of patients showed marked improvement (or complete normalization) in their peripheral blood counts. Bone marrow responses, however, were noted only in 51.6% of cases [35]. Other treatment options for AIMF include azathioprine, cyclosporine, mycophenolate mofetil, and intravenous immunoglobulin [4, 35, 40]. Plasma exchange was not shown to be useful in the management of AIMF [4]. The resolution of peripheral cytopenias and marrow fibrosis after institution of corticosteroids also strengthened the diagnosis of AIMF in our case.

The parallel existence of AAHS and AIMF has only been reported once in the literature. Kondo et al. in 2015 presented a case of a 48-year-old female patient presenting with moderate leukopenia (3.1 × 10^9^/L), anemia (hemoglobin 99 g/L), elevated ESR (74 mm/h), elevated triglycerides (2.57 mmol/L), and hypocomplementemia [37]. ANA was high and anti-dsDNA was also elevated. Anti-SS-A antibodies were also detected. Serum ferritin and transaminases were, however, normal. Bone marrow aspiration, which was almost a dry tap, revealed hemophagocytosis. Bone marrow trephine biopsy revealed myelofibrosis. Additional laboratory findings include normal serum IL-1*β* (<10 pg/mL) but markedly elevated TGF-*β* (31.2 ng/mL), TNF-*α* (40 pg/mL), and IL-6 (58.5 pg/mL). She was diagnosed with SLE complicated with hemophagocytosis and myelofibrosis. She was given prednisolone, after which symptoms and laboratory findings gradually improved. Repeat bone marrow aspiration and biopsy before discharge revealed improvement in hemophagocytosis and myelofibrosis.

**Figure 4:**
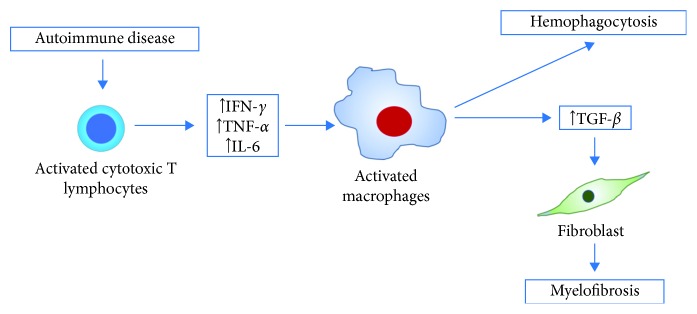
Possible pathogenesis of coexisting hemophagocytosis and myelofibrosis in SLE. The state of immune activation in SLE leads to excessive production of cytokines such as IFN-*γ*, TNF-*α*, and IL-6. TNF-*α* is known to induce activation of macrophages. AAHS occurs as activated macrophages engulf mature and hematopoietic stem cells and further contribute to cytokine production. TGF-*β*, which is a cytokine produced by activated macrophages, stimulates fibroblast proliferation leading to AIMF.

In our case and in the report of Kondo et al., the improvement of hemophagocytosis and myelofibrosis with the use of immunosuppressive treatment underscores immune dysregulation as the most plausible central pathogenetic mechanism [37]. SLE is generally characterized by loss of self-tolerance with activation of autoreactive T and B cells leading to autoantibody- and cytokine-mediated tissue injury [41]. The predominantly activated immune state in SLE and overwhelming cytokine release (e.g., IFN-*γ*, TNF-*α*, and IL-6) results in excessive macrophage activity. These activated macrophages engulf mature and hematopoietic stem cells, leading to the phenomenon of hemophagocytosis and further contribute to cytokine production [6, 9]. An important cytokine produced by macrophages is TGF-*β*, which is generally implicated in the pathogenesis of bone marrow fibrosis [42]. TGF-*β* increases the synthesis of collagen in addition to the deposition of fibronectin, proteoglycans, and tenascin [43]. The hypercytokinemia seen in AAHS leads to the production of large amounts of TGF-*β,* which stimulates fibroblast proliferation leading to AIMF [37]. Kondo demonstrated TNF-*α* and TGF-*β* elevations in his report which highlight the possible involvement of these cytokines in the pathogenesis of concurrent hemophagocytosis and myelofibrosis [37].  Figure 4 summarizes the proposed mechanism for the parallel existence of AAHS and AIMF. Additional studies, however, are needed to elucidate the exact mechanisms leading to hemophagocytosis and myelofibrosis in SLE.

## 4. Conclusion

In this report, we presented a rare case of SLE with concomitant AAHS and AIMF successfully treated with corticosteroid therapy. Hemophagocytosis and myelofibrosis, although uncommon, are possible initial manifestations of SLE and thus should be included in the differential diagnosis of cytopenias in SLE. Thorough clinical assessment and extensive microscopic examination of the bone marrow are important in diagnosing these conditions which are potentially life-threatening. Timely initiation of corticosteroid therapy is important in the treatment of both AAHS and AIMF in SLE. Finally, it must be emphasized that the bone marrow is an important site of target organ damage in SLE, and evaluation of cytopenias in SLE must take this into consideration.
